# Why the United States needs a multifaceted definition of health

**DOI:** 10.1093/haschl/qxad048

**Published:** 2023-09-19

**Authors:** Kevin Fiscella, Ronald M Epstein

**Affiliations:** Department of Family Medicine, University of Rochester Medical Center, Rochester, NY 14620, United States; Department of Family Medicine, University of Rochester Medical Center, Rochester, NY 14620, United States; Department of Oncology, University of Rochester School of Medicine and Dentistry, Rochester, NY 14620, United States; Department of Medicine (Palliative Care), University of Rochester School of Medicine and Dentistry, Rochester, NY 14620, United States

**Keywords:** whole health, population health, health equity, definition

## Abstract

How health is conceived and operationalized is an unrecognized contributor to poor health outcomes in the United States. The United States lacks an explicit definition of health, yielding a de facto, implicit biomedical definition in research and in health care that contrasts with how many people define health for themselves. This biomedical conceptualization has led to the development of lifesaving drugs, vaccines, and procedures, but has also resulted in critical underinvestment in people across their lives, beginning in early childhood, in behavioral, environmental, and social determinants. This underinvestment across the entire lifespan in people's health traps the United States in a vicious cycle of chronic disease and unsustainable health care costs. A movement towards holistic definitions of health represents an escape by defining health in more meaningful terms that reflect people's early development, agency, functioning, adaptive capacity, well-being, and lifelong development—that is, the capability for every person to thrive. Adopting and implementing a multifaceted, holistic health definition by federal research and health agencies could transform and humanize health in the United States and advance health equity.

Few things are as important to us as our health and that of those we love. For most people, health means more than the absence of disease.^1^ People see health as the capability to achieve physical, mental, and social well-being through the ability to identify and realize aspirations, satisfy needs, and change or cope with their environment.^2^ By many indicators, health in the United States is declining precipitously.^3,4^ The United States lags behind other wealthy countries in life expectancy^5^ and infant^6^ and maternal mortality;^7^ COVID-19 accelerated the US decline in health.^8^

## Implicit biomedical definition of health

The current health crisis is, in part, due to a lack of clarity regarding the meaning of health.^9^ Despite its importance in our lives, there is no national definition of health. Even federal agencies dedicated to the improvement of health do not explicitly define what they seek to improve. Searching glossaries, websites, media relations, and publications by the National Institutes of Health (NIH)^10^ and the US Department of Health and Human Services (HHS)^11^ or the Centers for Medicare and Medicaid Services (CMS)^12^ failed to identify a definition of health.

The absence of a US definition of health defaults to a flawed biomedical, disease-based model that is at odds with how most define their health.^1^ The NIH's core mission is basic biomedical research,^13^ with over half^14^ of its $50 billion annual budget^15^ for basic (preclinical) research. Similarly, health care is disease focused, with reimbursement based on billing for “procedures” for diagnosing and treating diseases categorized according to 1 of 71 000 categories.^16^ Of the $4.3 trillion^17^ spent annually on health care (>18% of the US Gross Domestic Product), 90% is for chronic and mental diseases.^18^

The biomedical model has undeniable benefits. Biomedical investments drive clinical practice and research and have yielded awe-inspiring treatments for selected diseases (eg, HIV,^19^ hepatitis C infections,^20^ several cancers,^21^ and genetic disorders^22^).

Despite its prowess in selected areas, the biomedical model has an Achilles heel. Its laser-like focus on mechanisms and developing patentable single interventions ignores people's humanity, their whole selves as holistic beings, and their potential for growth and development. This narrow focus leaves little to address this hidden human potential for health and human development in the holistic sense—that is, well-being, daily functioning, resilience, and capability for a meaningful and flourishing life. This model traps the United States in a vicious cycle of spiraling chronic disease and spending. Arguably, we know more about editing human genes than we know about pragmatic strategies for optimizing human health. Suboptimal funding for research on human development and potential that is outside the dominant model misses opportunities to discover how to optimize all people's and communities’ health, do so equitably, and align these discoveries into the structure and goals of health care organizations to optimize human development and health holistically.

## A multifaceted, holistic definition of health

To help people thrive and be healthy, federal organizations need to define health in multifaceted, “person-centered,” and “people-centered” terms and describe how their organizational mission is aligned with their definition. A “person-centered” definition^23^ considers positive aspects of health, meaning, well-being, and capabilities as well as the prevention and treatment of specific diseases. “People-centered health services”^24^ are explicitly designed to place persons, people (eg, families, caregivers, etc), and the community at the center of the care model. A multifaceted, holistic definition of health could direct needed innovations for research, health care, and health equity during an era when innovation in research seems to be slowing^25^ and when US health care fails to deliver either value or equity.^26^

A multifaceted definition must also consider the developmental nature of health—that is, that reflects being and becoming healthy. In 1948, the World Health Organization (WHO) Constitution defined health: “Health is a state of complete physical, mental and social well-being and not merely the absence of disease or infirmity.”^27^ A 21st-century holistic definition recognizes health as a multifaceted, dynamic, developmental, and adaptive concept encompassing physical, mental, emotional, social, and spiritual aspects of people's current and future lives.

Health is dynamic. Health begets health. Health is continuously molded by the interaction of genetic, environmental, behavioral, and social elements, coupled with personal experiences. Key life stages (particularly in early childhood) and events may alter peoples’ health and health trajectories, including their capability for health, with multilevel interventions potentially mitigating adverse trajectories.^28^ Health capability is defined as the ability of a person to be and become healthy.^29^ It is holistic and multidimensional, reflecting capacities within the person in conjunction with their social and environmental context to achieve and maintain a state of health.^29^

Fundamental to this definition is that is a dynamic “meta-capability” that reflects continuous interaction between people's biological endowments, acquired abilities, and facilitating or hindering social and environmental contexts, which critically enables people to grow, develop, learn, adapt, thrive, love, and be loved in ways that optimize current and future states.^30^ People cherish both the intrinsic and instrumental value of health—that is, the experience of well-being and function in addition to the ability to satisfy important goals.^31^

The WHO definition must be expanded to consider social conditions and resources that optimize not only function and well-being but also development (ie, continuous growth in capability to be healthy). The emerging science of biological aging is discovering epigenetic and other biomarkers for “accelerated aging” that reflect the cumulative, hidden impact of social conditions.^32^ These biomarkers are significant for their potential to reflect future health—that is, premature morbidity and mortality and result from adverse child experiences; deleterious environmental exposures; physical, mental, and social trauma; inadequate resources; despair; maladaptation; and unfavorable trajectories in human development and life courses.

This definition of health suggests consideration of a broader meaning of the term “social determinants of health” (ie, consideration of fundamental social conditions that impact lifelong growth and development), suggesting the critical need to optimize social conditions—that is, address inequities in resources and structural racism, and deploy interventions that optimize human agency, function, development, well-being, and health capability, and doing so in ways that promote health equity. This conceptualization diverges radically from the medicalized version of social determinants of health involving screening and referral.

## Benefits of a national, holistic, multifaceted definition of health

The potential benefits for the HHS, NIH and CMS in adopting a holistic definition of health are profound. First, this health definition would improve the alignment of their missions with the societal goal of improving health defined, holistically and developmentally. It offers opportunities to discover strategies that inform policies to optimize health and implement them in health care and in partnership with communities. Alignment behind a common holistic, life course definition of health that embraces equity could help reverse declining national life expectancy^4^ and excess deaths^33^ relative to peer countries. The health of people is intertwined with important others and with their communities. Arguably, optimizing health defined holistically and equitably would help address stark racial and ethnic disparities in maternal and child health within the United States, between states in the United States, and between the United States and peer countries.^7,34^

Second, such a definition is critical to health equity. It expands the concept of health equity beyond equity in the absence of disease to equity in growth, development, and capability for health and physical, mental, and social well-being. This definition creates opportunities to address inequities more profoundly as envisioned in the Gross (Child) Health Potential^35^ and points to collective responses to effectively operationalize it meaningfully.

The Centers for Disease Control and Prevention defines health equity as “… the state in which everyone has a fair and just opportunity to attain their highest level of health. Achieving this requires ongoing societal efforts to address historical and contemporary injustices; overcome economic, social, and other obstacles to health and health care; and eliminate preventable health disparities.”^36^ Rather than investing only in the diagnosis and treatment of disease, this definition suggests continuous investment in people, beginning by investing in socially disadvantaged future mothers, families, communities, and particularly children during critical phases. Consideration of the factors that affect inequities in human development and capability is fundamental to ensuring equity in health in the fullest sense.

Third, a clear definition of health could better operationalize “value” in value-based payment beyond disease-based process measures.^37^ The benefits of paying for value are constrained by how health is defined, measured, and operationalized. Measures such as cancer screening or avoidable hospitalizations are inadequate alone for health. Conversely, holistic measures of health could be dynamic people- and community-focused and built on child developmental capabilities,^38^ health capabilities,^29^ Whole Person Health Scores,^39^ community health measures,^40^ and other measures that matter to people.^41^

Fourth, a holistic definition can influence how health research is conducted, and offers the possibility for generating pragmatic interventions and informing policies that address behavioral, developmental, environmental, and social determinants of health.^42^ Such a definition could help create a complementary translational research pathways that focuses on discovery, adaptation, and implementation of holistically oriented interventions that address this goal.^42^

Last, a whole health definition centers health care around people, as reflected in the tagline for the Department of Veteran Affairs’ (VA's) Whole Health initiative, “What matters to you, not what is the matter with you,”^43^ aligns the values and culture of health care organizations with people-centered care while supporting the well-being and resiliency of health care personnel. As Prah Ruger^44^ notes, Aristotle argued that promotion of human flourishing is the end goal of political activity. She quotes Aristotle: “It belongs to the excellent legislator to see how a city, a family of human beings … will share in the good life and in the happiness that is possible for them”—and suggests that his writing provided a foundation for the concept of health capability.^44^

The implicit adoption of a dehumanizing, exclusively disease-based definition of health is a major obstacle to addressing the national health crisis in the United States. The time is now for CMS, and NIH, to support a multistakeholder process to adopt and refine multifaceted holistic definitions of health that recognize the power of investing in people's health potential across their lives by enabling them to direct their health care and optimize their—and their community's—health.

In concert with relevant federal agencies and diverse communities, we need to kindle national action regarding the full and complete meaning of health, its facets, and its early origins and development,^28^ and how to operationalize a holistic health model relevant to all Americans. This requires addressing challenges raised by entrenched institutional cultures, structures, and reward systems. Implementing such an approach in research and health care requires vision, leadership, and resolve, recognizing that the current system is utterly failing. Success requires community participation and national leadership and action.^9^

## Next steps

The VA and other national organizations have taken important first steps by defining health in broader terms and enacting programs concordant with their new emphasis on whole health.^45^ These efforts represent just the beginning. There is an urgent need to expand the VA's Whole Health model to explicitly incorporate health equity and address the limitations of just considering well-being and current disease states. Optimizing population health and health equity requires broader consideration of the developmental nature of health and balancing imperatives, extending from the beginning to the end of life, for well-being in the present, and longevity and thriving in the future, with equity as a core principle. There is an urgent need for an actionable, clear, multifaceted, and holistic definition of health, reflecting what matters the most to the people and communities we serve and that recognizes the salience of human development and capability for health across the lifespan to reverse the crisis of declining health in the United States and mitigate longstanding health inequities.

## Supplementary Material

qxad048_Supplementary_Data
